# The m.15043G > A *MT-CYB* variant is not a pathogenic mtDNA variant

**DOI:** 10.1016/j.jns.2020.116950

**Published:** 2020-10-15

**Authors:** Charlotte L. Alston, Emma L. Blakely, Robert McFarland, Robert W. Taylor

**Affiliations:** a Wellcome Centre for Mitochondrial Research, Newcastle University, Framlington Place, Newcastle upon Tyne, NE2 4HH, UK; b Translational and Clinical Research Institute, Faculty of Medical Sciences, Newcastle University, Framlington Place, Newcastle upon Tyne, NE2 4HH, UK; c NHS Highly Specialised Service for Rare Mitochondrial Disorders, Royal Victoria Infirmary, Newcastle upon Tyne Hospitals NHS Foundation Trust, Queen Victoria Road, Newcastle upon Tyne, NE1 4LP, UK

Dear Editor,

We read with interest the article by Ghosh and colleagues describing an adolescent male who presented with primary hypoparathyroidism and extensive neurological involvement [2]; the authors conclude that the mitochondrial DNA (mtDNA; NC_012920.1) variant, m.15043G > A within *MT-CYB* is pathogenic and responsible for the patient's neurological presentation.

We wish to briefly and unequivocally refute this conclusion; we provide supporting evidence that the m.15043G > A mtDNA variant is benign and does not underlie this patient's presentation.

## 1 The m.15043G > A variant is far too common to be pathogenic

ACMG guidelines are regarded as the benchmark for variant classification [5] and when applied to the synonymous m.15043G > A; p.Gly99Gly variant demonstrate a neutral effect, primarily because of its frequency. Stand-alone criteria BA1 can be applied given that it is present in >5% of the population; m.15043G > A is a single nucleotide variant comprising the backbone of Haplogroup M and is present in 23.6% of the MITOMAP dataset (a compendium of 51,192 mtDNA genome sequences) [4]. Moreover, we have observed the m.15043G > A *MT-CYB* variant in approximately 10% of our own patient cohort (>2000 full mtDNA sequences) including a number of patients with an alternative, established genetic diagnosis of mitochondrial disease. In all instances, the m.15043G > A variant occurs as a homoplasmic variant, consistent with its benign nature - the vast majority of pathogenic mtDNA variations are heteroplasmic, bar a few exceptions [3]. Segregation studies for pathogenic mtDNA variants should be performed, where possible, using non-invasively sourced maternal DNA samples (i.e. EDTA-blood, urinary sediment and buccal epithelium) and mtDNA variant heteroplasmy levels correlated with maternal clinical status and any relevant family history; unfortunately, no segregation studies were performed nor is a family history documented.

## 2 There is no evidence of perturbed cytochrome *b* activity

The authors claim that “the homoplasmic mtDNA variant m.15043G > A is pathogenic and perturbs cytochrome-b activity”. No evidence is provided in support of this, and no muscle biopsy was available in which to assess mitochondrial complex III activity.

## 3 Conclusion

While a number of the clinical features described in this case are compatible with mitochondrial disease (sensorineural hearing loss, bilateral ptosis and elevated blood and CSF lactates) others can be attributed to chronic hypoparathyroidism – T1 hyperintense signal abnormalities of basal ganglia, reversible cognitive impairment, muscle weakness and choreiform limb movements. Nevertheless, assuming a mitochondrial disease diagnosis is correct, analysis of the available data underlies our opinion that the synonymous m.15043G > A variant is not pathogenic and cannot therefore be the cause of the phenotype described by the authors. This report highlights the importance of full and thorough interpretation of patient genetic data to ensure that the correct diagnosis is ascribed [1]. Moreover, it validates the clinical utility of a diagnostic biopsy and the use of patient material for the functional validation of genetic findings as the inadequate assessment of pathogenicity has the potential to result in patient misdiagnosis and mismanagement. Given the dual genetic control of mitochondrial function and large number of possible Mendelian-mitochondrial aetiologies, a gene agnostic whole exome or whole genome sequencing approach would seem to be appropriate to ascertain a molecular diagnosis in the absence of a pathogenic mtDNA variant [6].

## Funding

Work in our laboratories is supported by the Wellcome Centre for Mitochondrial Research (203105/Z/16/Z), the Medical Research Council (MRC) International Centre for Genomic Medicine in Neuromuscular Disease, the Mitochondrial Disease Patient Cohort (UK) (G0800674), the Lily Foundation, the UK NIHR Biomedical Research Centre for Ageing and Age-related disease award to the Newcastle upon Tyne Foundation Hospitals NHS Trust, the MRC/EPSRC Molecular Pathology Node and the UK National Health Service Highly Specialised Service for Rare Mitochondrial Disorders, CLA is supported by the 10.13039/501100000272National Institute for Health Research (NIHR Post-Doctoral Fellowship, PDF-2018-11-ST2-021). The views expressed in this publication are those of the author(s) and not necessarily those of the NHS, the National Institute for Health Research or the Department of Health and Social Care.

## Author contribution

CA: design, literature search, discussion, first draft, revision; ELB: critical comments, literature search, revision; RM: critical comments/revision; RWT: critical comments/revision.

All authors have read the journal's position on issues involved in ethical publication.

## Declaration of Competing Interest

There are no conflicts of interest.
